# Evolutionary Interplay between Symbiotic Relationships and Patterns of Signal Peptide Gain and Loss

**DOI:** 10.1093/gbe/evy049

**Published:** 2018-03-19

**Authors:** Peter Hönigschmid, Nadya Bykova, René Schneider, Dmitry Ivankov, Dmitrij Frishman

**Affiliations:** 1 Department of Bioinformatics, Technische Universität München, Wissenschaftszentrum Weihenstephan, Freising, Germany; 2 Institute for Information Transmission Problems (Kharkevich Institute), RAS, Moscow, Russia; 3 Institute of Science and Technology Austria, Klosterneuburg, Austria; 4 Laboratory of Bioinformatics, RASA Research Center, St. Petersburg State Polytechnical University, Russia

**Keywords:** comparative genomics, molecular evolution, sequence analysis, signal peptides

## Abstract

Can orthologous proteins differ in terms of their ability to be secreted? To answer this question, we investigated the distribution of signal peptides within the orthologous groups of *Enterobacterales*. Parsimony analysis and sequence comparisons revealed a large number of signal peptide gain and loss events, in which signal peptides emerge or disappear in the course of evolution. Signal peptide losses prevail over gains, an effect which is especially pronounced in the transition from the free-living or commensal to the endosymbiotic lifestyle. The disproportionate decline in the number of signal peptide-containing proteins in endosymbionts cannot be explained by the overall reduction of their genomes. Signal peptides can be gained and lost either by acquisition/elimination of the corresponding N-terminal regions or by gradual accumulation of mutations. The evolutionary dynamics of signal peptides in bacterial proteins represents a powerful mechanism of functional diversification.

## Introduction

Protein function is not set in stone—it can undergo both gradual and drastic changes due to a variety of evolutionary events, including mutations, insertions, deletions, and duplications. Early on it was noted that proteins sharing the same structural fold can have vastly divergent functional roles (Devos and Valencia 2000). Although functional equivalence of orthologs is often assumed, recent assessments indicate a rather low degree of functional similarity between pairs of orthologous genes (Altenhoff et al. 2016), even when they share very high overall sequence identity (Nehrt et al. 2011). Specific aspects of proteins function may vary between orthologs significantly, including enzymatic specificity (Rost 2002) and protein interaction sites (Aloy et al. 2003). Local molecular determinants of protein function, such as phosphorylation sites, as well as entire protein domains, can be gained and lost in the course of evolution.

Although the evolutionary dynamics of enzymatic and binding activities has been extensively studied, functional shifts associated with the evolution of cellular targeting signals have received much less attention, and most of the work done so far focused on the sequence diversity of eukaryotic signal peptides, mitochondrial targeting signals, and chloroplast transit peptides (Williams et al. 2000; Doyle et al. 2013; Fukasawa et al. 2014). In particular, differences in the evolutionary rates between intra- and extracellular proteins have been reported for mammals and yeast (Julenius 2006; Liao et al. 2010), and shown to depend on tissue-specific gene expression (Winter et al. 2003). In bacteria, the majority of the secreted proteins (96% in *Escherichia coli*; Tsirigotaki et al. 2017) are translocated across the cytoplasmic membrane in a Sec-pathway-dependent manner and possess cleavable signal peptides—short sequence segments of 20–30 amino acids in length, which act as targeting signals (Heijne 1990). Signal peptides exhibit a tripartite structure, consisting of a positively charged N-terminal region, a central hydrophobic region, and a polar C-terminal region, which contains a three-residue cleavage motif recognized by the signal peptidase I (von Heijne 1985, 1990). The limits of sequence variation within signal peptides have been extensively studied (Heijne 1985; Hegde and Bernstein 2006) and a large number of nonconventional taxon-specific sequences have been identified by proteogenomic experiments (Payne et al. 2012). However, all these studies were primarily aimed at understanding the minimal sequence requirements of the signal recognition machinery and did not consider evolutionary effects associated with elimination or acquisition of signal peptides.

Given the importance of signal peptides for protein sorting and localization it is no wonder that they constitute an important element of protein and genome annotation. Early analyses of completely sequenced genomes suggested that around 20% of proteins are secreted in a typical bacterium, such as *Haemophilus influenzae* (Nielsen et al. 1997) or *Escherichia coli* (Käll et al. 2004). More recently these estimates have been revised due to both improved accuracy of bioinformatics predictions (Petersen et al. 2011) and the availability of proteogenomics data (Gupta et al. 2007; Venter et al. 2011), and for the best studied bacterium *Escherichia coli* they currently converge to 10% of proteins possessing a signal peptide (Ivankov et al. 2013). The size and the composition of the secretome are highly informative for understanding organism’s physiology. An important driving force for functional divergence in bacteria is constituted by environmental variation and the ensuing changes of lifestyle. In general, pathogenic and nonpathogenic species would be expected to secrete different proteins (Trost et al. 2005), but a recent study (Song et al. 2009) failed to establish any connection between pathogenicity and the secretome size. A positive correlation between the percentage of secreted proteins and the number of genes in the Gram-negative, but not in the Gram-positive organisms, was reported.

Here, we present a comparative secretome analysis of *Enterobacterales*, focusing not only on the relative number of secreted proteins but also on the conservation of their ability to be secreted in relation to the bacterial lifestyle. In order to conduct this analysis, we integrated evolutionary trees of orthologous protein groups with signal peptide predictions and functional annotation. Parsimony analysis and sequence comparisons revealed a large number of signal peptide gain and loss events, in which signal peptides emerge or disappear among orthologous proteins in the course of evolution. We also attempted to shed light on the molecular mechanism leading to these events and their relationship to the symbiotic lifestyle of an organism. Our results indicate that signal peptide losses prevail over gains, an effect which is especially pronounced in the transition from the free-living or commensal to the endosymbiotic lifestyle. The disproportionate decline in the number of signal peptide-containing proteins in endosymbionts cannot be explained by the overall reduction of their genomes (Andersson and Kurland 1998). Signal peptides can be gained and lost either by acquisition/elimination of the corresponding N-terminal regions or by gradual accumulation of mutations.

## Materials and Methods

### Genomes, Orthologous Clusters, and Gene Ontology Terms

The *Enterobacterales* order is a large and diverse group of Gram-negative bacteria within the class *Gammaproteobacteria*. Its taxonomic tree has been recently updated and refined (Adeolu et al. 2016). This group, to which the best studied model organism *Escherichia coli* also belongs, contains bacteria occupying a variety of habitats and involved in diverse kinds of symbiotic relationships. The taxonomic identifiers of these organisms were extracted from the NCBI (National Center for Biotechnology Information) taxonomy database (Wheeler et al. 2007) in November 2016. The corresponding genomes were downloaded either from the ENA (European Nucleotide Archive) (Pakseresht et al. 2014) or the EnsemblGenome database (Kersey et al. 2016). *Enterobacterales* clusters of orthologous groups (COGs) with associated GO-terms were retrieved from the OMA orthology database in June 2016 (Altenhoff et al. 2015). The resulting data set contains 626,680 proteins from 153 distinct species, of which 557,556 proteins are mapped onto 24,837 orthologous clusters.

### Evolutionary Trees

Evolutionary trees for all clusters were built with PhyML 3.0 (Guindon et al. 2010) using multiple sequence alignment (MSA) of cluster members as input. MSAs were computed by Clustal Omega (Sievers et al. 2014) with the default parameters. As PhyML only produces unrooted trees, which do not provide any information about the direction of evolution, we rooted the tree using the midpoint rooting method, which takes the longest distance between two leafs in the tree, and inserts the root at the exact midpoint between them. Since at least three proteins are required to calculate an evolutionary tree, clusters with one or two members were not considered.

### Signal Peptide Data

Signal peptides were identified in the *Enterobacterales* gene products based on three data sources with a different degree of confidence. First, signal peptides were predicted by the latest and most accurate version of the SignalP (SignalP 4.1; Petersen et al. 2011) software with all default parameters using the Gram-negative model. In addition, signal peptides were predicted by Phobius (Käll et al. 2004, 2005), which, in contrast to SignalP, returns discrete predictions rather than scores.

As we focus on Sec-mediated protein secretion, we used TatP (Bendtsen et al. 2005) to remove COGs containing proteins utilizing the twin-arginine translocation (Tat) pathway.

Results of these three methods were combined to derive a consensus prediction with four possible outcomes: 1) twin-arginine signal peptide predicted by TatP (leads to rejection of the entire COG), 2) Sec signal peptide reliably predicted (positive SignalP and Phobius predictions), 3) absence of a Sec signal peptide reliably predicted (negative predictions by both SignalP and Phobius), 4) discordant Sec signal peptide assignments by SignalP and Phobius (protein gets discarded).

In order to find COGs with contradicting signal peptide assignments, that is, those clusters where signal peptide gain and loss events happened, they were subdivided into positive, negative, or mixed clusters containing only positive, only negative, or both positive and negative predictions.

### Assignment of Symbiont Status to bacteria

We manually annotated organisms according to their lifestyle as either symbiotic or free-living bacteria. The symbionts were further subdivided into either endosymbionts or commensals. In the former relationship both partners benefit from the interactions, whereas in the latter relationship, only one partner gains benefits, whereas the other is affected neither in a positive nor in a negative way. Out of the 153 genomes, 33 (21.6%) were classified as symbionts—12 of them as commensals and 21 as endosymbionts.

### Evolutionary Model and Parsimony Analysis

We seek to identify signal peptide gain and loss events in the evolutionary history of *Enterobacterales* orthologous families. The input data for this analysis are constituted by the evolutionary tree of the extant protein sequences in each family and the predicted signal peptide states of the exterior nodes (leafs). The latter can be expressed as a presence/absence dichotomy. Signal peptide states for the internal nodes are reconstructed using the parsimony method by Fitch (Fitch 1971), which essentially assigns the signal peptide states such that the number of state transitions in the tree is minimal. Given the tree, the inferred states at the internal nodes and the states at the leaf nodes, where a negative state (0) and a positive state (1) indicate the absence and the presence of a signal peptide, respectively, a gain event corresponds to the transition from a negative state to a positive state at some branch of the tree, whereas the loss event corresponds to the opposite transition.

We conducted this standard parsimony analysis for all protein families with contradicting signal peptide assignments between individual family members (“mixed” families). Only discrete signal peptide data (i.e., presence or absence) were considered to infer ancestral states. Tentative signal peptide loss events resulting from the first round of parsimonious reconstruction were verified by comparative genomics and used to conduct a gene start correction procedure, as described in the next section. Subsequently a second parsimony analysis was conducted to infer the final signal peptide states for all internal nodes of the trees and to estimate the effect of the start correction procedure.

Along with the second parsimony analysis for signal peptides, the Fitch algorithm was also applied to the symbiont states. The leaf nodes (organisms) were assigned either state 2 if the organism lives in a commensal relationship, state 1 if it lives in an endosymbiotic relationship, or state 0 if it is a free-living bacterium. After inferring the ancestral states using the Fitch algorithm, transition events between all three states along the evolutionary tree were derived.

### Gene Start Correction

Based on the results of the initial parsimony analysis, we investigated the possibility of spurious gain or loss events caused by incorrect gene starts. All trees containing leaves (extant proteins) with contradicting signal peptide assignments, that is, the mixed clusters, were traversed. In case a leaf was predicted not contain a signal peptide both by SignalP and Phobius, a set of proteins with alternative start positions (considering the ATG, GTG, and TTG start codons) was constructed for this specific protein. The size of the sequence neighborhood scanned up- and downstream for an alternative gene start was determined based on the MSAs calculated in the first round of the parsimony analysis as follows. The position of the first residue in the MSA of each protein without a signal peptide prediction was compared against all first residue positions of proteins with signal peptides. The maxima of these distances in both directions, up- as well as downstream (plus another 30 residues in each direction) were used as search space. Subsequently SignalP, Phobius, and TatP predictions were made for the N-termini of these new proteins. A start position was chosen dependent on the prediction outcomes in the following order of priority: 1) positive TatP prediction, resulting in the deletion of the entire COG, 2) reliable positive or negative prediction (agreement between SignalP and Phobius), 3) disagreement between SignalP or Phobius, resulting in the deletion of the protein, or 4) gene start left unchanged, that is, the reliable negative prediction remains valid. In cases where multiple gene starts lead to a reliable positive prediction, the one with the highest SignalP prediction score was chosen.

### Functional Annotation of Protein Groups

To calculate the enrichment of GO terms in the positive, negative, and mixed groups, GO annotations assigned to each individual protein were supplemented with their parent terms according to the GO tree. Searching for enriched terms was then achieved by applying a one-sided Fisher’s exact test to each term in each group using the occurrence frequency of the term in all groups as a background model. A similar analysis was performed solely on the proteins in the mixed groups in order to understand the functional implications associated with the gain and loss of signal peptides.

### Assignment of Taxonomic Positions to Signal Peptide Gain and Loss Events

For each event reconstructed on the evolutionary tree by the method described earlier, we first determined all children leafs of the node where the event happened, and the species, genus, family, and order of each of the corresponding organisms. We then identified the minimal common taxonomy rank for this resulting group of genomes using the NCBI taxonomy tree. As a result, the taxonomic rank of that event could be determined.

### Discrimination Score

For each COG *g* a discrimination score *d(a, b, g)* was calculated as:

$$d\left(a,\;b,\;g\right)=\frac{a_{sp}-a_{\overset{-}{sp}}}{a_{sp}+a_{\overset{-}{sp}}}-\frac{b_{sp}-b_{\overset{-}{sp}}}{b_{sp}+b_{\overset{-}{sp}}}$$

where *a* and *b* are two lifestyles to be compared, that is, free-living bacteria, commensals or endosymbionts, whereas $a_{sp}$ and $a_{\overset{-}{sp}}$ are the numbers of proteins associated with the lifestyle *a* and $b_{sp}$ and $b_{\overset{-}{sp}}$ are the numbers of proteins associated with the lifestyle *b* with and without signal peptide in COG *g*. The result ranges from −2 to 2, where more negative values mean that in this group bacteria of type *a* tend to have fewer signal peptides than bacteria of type *b*, whereas a more positive value means the opposite. In addition, the closer the result is to the two extrema −2 and 2, the more discriminating the possession of a signal peptide is for separating lifestyles *a* and *b* in a particular group *g*, whereas values close to zero can be considered as indecisive.

## Results and Discussion

### Signal Peptides in the *Enterobacterales* Order

We conducted a comprehensive analysis of *Enterobacterales* secretomes based on bioinformatics predictions. Out of 626,680 gene products encoded in 153 *Enterobacterales* genomes derived from the OMA orthology database, 52,902 (8.4%) were identified as containing reliable signal peptides based on the intersection of positive SignalP, positive Phobius and negative TatP predictions, respectively, whereas 518,174 (82.7%) proteins were determined to be reliable negatives. The remaining 55,604 (8.9%) cases consist of 52,050 (8.3%) discordant predictions (51,787 predicted positive only by Phobius, 263 only by SignalP), and 3,554 (0.6%) twin-arginine signal peptides predicted by TatP. The average percentage of proteins with signal peptides per genome in our data is 7.7 ± 2.6%; the percentage scales roughly linearly with the genome size, increasing from 0.2% in *Riesia pediculicola USDA* over 10.1% in the *Escherichia coli K12/MC4100/BW2952* to 10.7% in a yet unclassified *Enterobacteriaceae* bacterium (supplementary fig. S1*A*, Supplementary Material online). The *Escherichia coli* annotation is thus in line with our previous estimate (10%) of the secretome size for this genome (Ivankov et al. 2013).

### Occurrence of Signal Peptides in *Enterobacterales* COGs

**Fig. 1.—:**
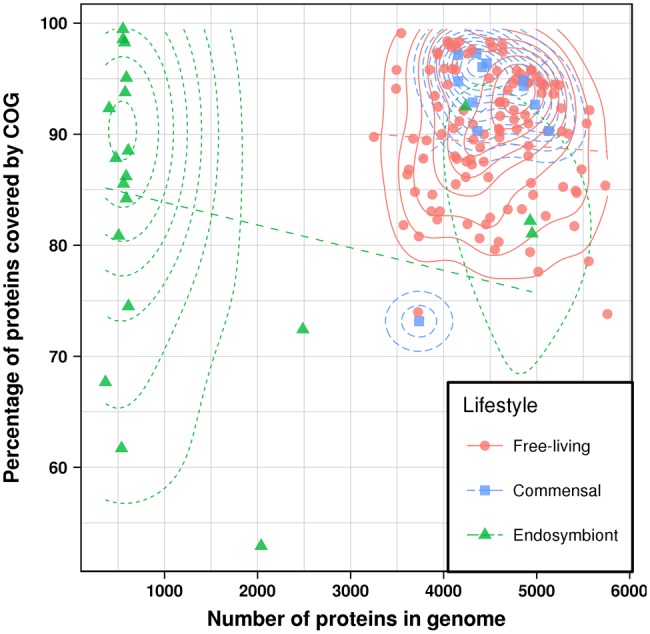
Number of proteins in a genome versus the percentage of proteins that are members of a COG. In addition to the raw values, the two-dimensional density and a linear fit (dashed lines) for each lifestyle is shown.

In total, 557,556 of the 626,680 proteins (89.0%) belong to 24,837 COGs with at least three members. On an average 88.6 ± 8.7% of proteins in the species considered are covered by COGs—from 52.9% in *Hamiltonella defensa subsp. Acyrthosiphon pisum 5AT* to 99.5% in *Buchnera aphidicola subsp. Acyrthosiphon pisum Tuc7*. The average COG coverage of small genomes, consisting of <1,000 genes, tends to be similar (86.3%±11.2) to that of large genomes with >3,000 genes (89.2%±7.8) (fig. 1) (*P* = 0.5, Kolmogorov–Smirnov test). The former correspond to endosymbiotic genomes that are thought to retain only the most functionally important and evolutionary conserved genes. The size of the clusters is 22.4 on an average and ranges from three (4,767 clusters or 19.2%), which is the smallest possible size, to 153 (7 clusters or 0.03%), which is a cluster containing a protein from every organism (supplementary fig. S2, Supplementary Material online).

After removal of 1,893 COGs which either contained a positive TatP prediction or did not satisfy the minimum number of three members after the removal of discordant signal peptide predictions, 498,690 of the initial 626,680 proteins (79.6%) were left in the data set and mapped to a COG. The percentage of these COG proteins possessing a signal peptide does not significantly differ from the percentage of signal peptide containing proteins in the entire proteomes. The total amount of proteins assigned as having a signal peptide is 47,139 (9.5%), with 8.6 ± 2.8% on an average per genome. In addition, the dependence on the genome size is essentially the same (supplementary fig. S1*B*, Supplementary Material online).

We subdivided the remaining 22,944 COGs according to the signal peptide assignments present in a cluster as described in the Materials and Methods section. This resulted in 20,363 negative clusters (88.8%), containing only proteins without signal peptides, 1,507 positive clusters (6.6%), containing only proteins with signal peptides, and 1,074 mixed clusters (4.7%), containing proteins both with and without signal peptides (see table 1). The mixed clusters can be assumed to contain those proteins that changed their cellular localization at least once in their evolutionary history, but could also result from wrong gene start annotation or wrong signal peptide assignments.

**Table 1:** Statistics on Clusters and Events for the Two Rounds of Parsimony Analysis Before and After the Gene Start Correction Procedure

Parsimonyround	Clusters				Events			
	Negative	Positive	Mixed	Total	Gain	Loss	Uncertain	Total
**1**	20,363 (88.8%)	1,507 (6.6%)	1,074 (4.7%)	22,944	325 (13.5%)	1,235 (51.2%)	852 (35.3%)	2,412
**2**	20,363 (89.0%)	2,087 (9.1%)	440 (1.9%)	22,890	83 (11.6%)	288 (40.2%)	346 (48.3%)	717

Since we are primarily interested in gain and loss of signal peptides, mixed clusters were further examined in order to estimate the scale of annotation errors and determine the biological significance of evolutionary events.

### Parsimony Analysis and Gene Start Correction

We conducted a first round of the parsimony analysis of the signal peptide assignments for the “mixed” COG clusters as described in the Materials and Methods section, that is, using the Fitch algorithm. In total 2,412 events were revealed, including 325 gains (13.5%), 1,235 losses (51.2%), and 852 uncertain events (35.3%) where the state could not be resolved by parsimony (table 1). Signal peptide losses thus prevailed over gains significantly (almost 4-fold).

**Fig. 2.—:**
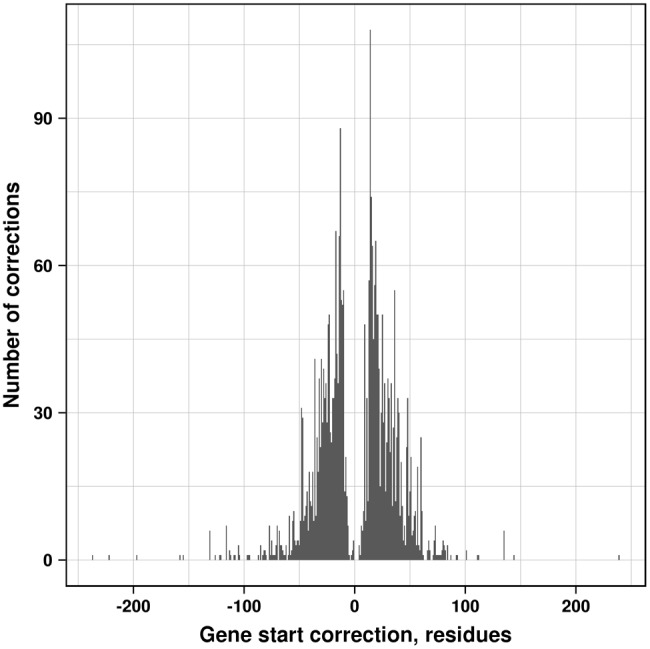
Distribution of gene start corrections, that is, the number of residues by which the protein sequence was extended (negative values) or truncated (positive values).

Following the first round of the parsimony analysis, we attempted to improve gene start annotation in order to minimize the number of false signal peptide events. Each protein without an assigned signal peptide was tested for a potential false negative prediction by shifting its gene start over a certain range determined by the signal peptide containing proteins in the same group (see Materials and Methods). After the gene start correction, the MSAs and the trees were recalculated using the updated sequences. Altogether, the correction procedure affected 3,005 proteins from 147 species, with the most affected genomes being *Cronobacter turicensis DSM 18703/LMG 23827/z3032* (127 corrections) and *Klebsiella pneumoniae subsp. pneumoniae ATCC 700721/MGH 78578* (54 corrections). In most cases gene starts underwent relatively small changes of their positions (fig. 2), with the average value of the absolute shift of +1.2 amino acids and the median value of +9; there were fewer corrections toward upstream gene start positions (1,450) then toward downstream positions (1,555).

**Fig. 3.—:**
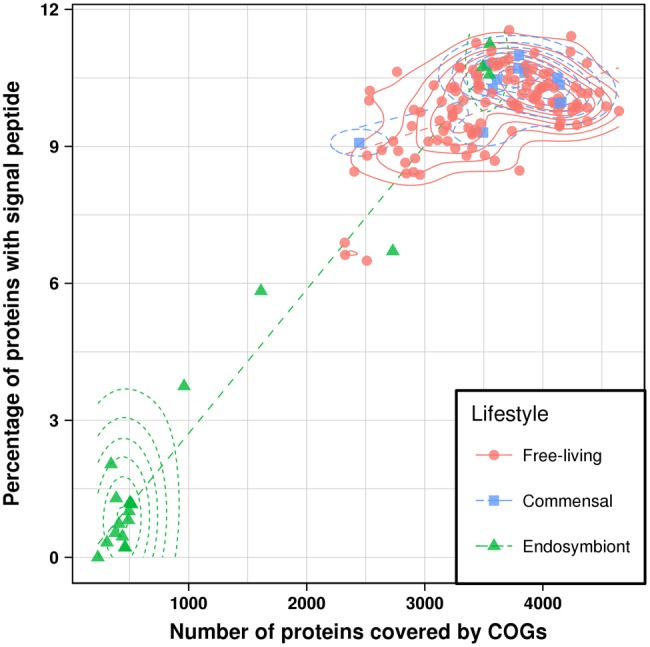
Number of proteins in a genome versus the percentage of proteins that possess a signal peptide after mapping of proteins to COGs, and after the gene start correction procedure. In addition to the raw values, the two-dimensional density and a linear fit (dashed lines) for each lifestyle are shown.

The gene start correction procedure led to changed signal peptide assignments for a number of proteins from “negative” to “positive,” the removal of proteins in which the correction revealed discordant predictions, and the deletion of certain mixed clusters due to either positive TatP predictions or fewer than three remaining proteins in the COG. Overall, only 29.7% of the events were kept compared with the first round of parsimony analysis, whereas 41.0% of mixed clusters remained (table 1). Based on these new assignments, we conducted a second round of parsimony analysis on the remaining 440 mixed clusters, which revealed 83 gain (11.6%), 288 loss (40.2%), and 346 uncertain events (48.3%) out of 717 events in total (table 1). Therefore, out of the 1,235 loss events from the first round of parsimony analysis, 947 events were recognized as false positives and 242 gain events were also eliminated. The ratio between gains and losses decreased only slightly, still being almost 4-fold. The percentage of signal peptides in our final data after mapping to COGs, removal of Tat signal containing groups and gene start correction is 48,817 out of 497,338 proteins (9.8%), with an average of 8.9 ± 2.9% per genome (fig. 3).

### Sequence Similarity of Secreted and Nonsecreted Proteins

**Fig. 4.—:**
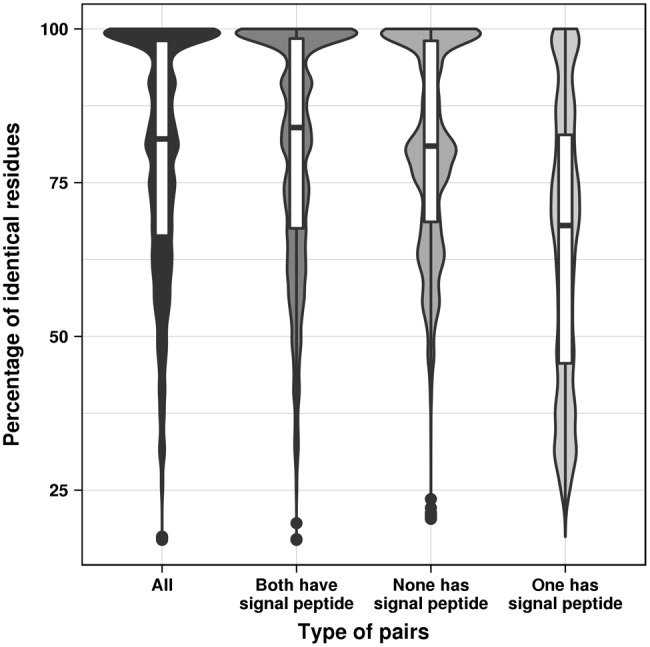
Comparison of sequence identity distributions between pairs of proteins where either both proteins have a signal peptide, or both have none, or only one protein has a signal peptide.

In order to find out whether the gain and loss patterns of signal peptides correlate with the evolutionary distance, we compared amino acid sequences of the proteins in the mixed groups. All possible pairwise sequence alignments were extracted from the MSA of each group and the pairwise sequence identity was calculated by dividing the number of identical residues by the length of the shorter sequence. We plotted the distributions of sequence identities for sequence pairs in which both, none, or only one sequence had a signal peptide (fig. 4). As expected, the mean of sequence identities for the pairs in which either no or both proteins possess a signal peptide (80.9%, 80.6%) is higher than for the pairs where only one protein gets secreted (64.8%), because in the latter case a smaller number of almost identical sequences occurs. If only protein pairs with a sequence identity <95% are considered, the three groups have much closer means (both have signal peptides: 71.5%, none has signal peptide: 73.4%, one has signal peptide: 59.9%).

### Evolutionary Mechanisms Leading to Gain and Loss of Signal Peptides

**Fig. 5.—:**
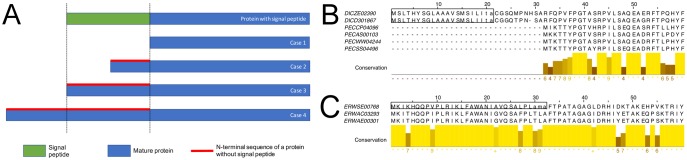
(*A*) The four possible cases for signal peptide gain and loss events. In proteins devoid of signal peptides the N-teminal sequence can be completely eliminated (case 1), shortened (case 2), have the same length (case 3), or be extended (case 4). Cases one and three are by far the most prevalent ones. (*B* and *C*) The first 60 positions in the MSAs of the proteins involved in a signal peptide gain event in “Pectinesterase” OMA group containing two Dickeya and four Pectobacteria (UniProt identifiers: C6CL61, Q47474 (reviewed), C6DIG6, Q6DAZ5, D0KDA3, P55743; reviewed) (*B*) and the gain event in the “putative Invasin” group containing three Erwinia species (UniProt identifiers: E3DHH7, D4I2A7, unknown) (*C*). Rectangles indicate signal peptides, with cleavage sites in lowercase letters.

**Fig. 6.—:**
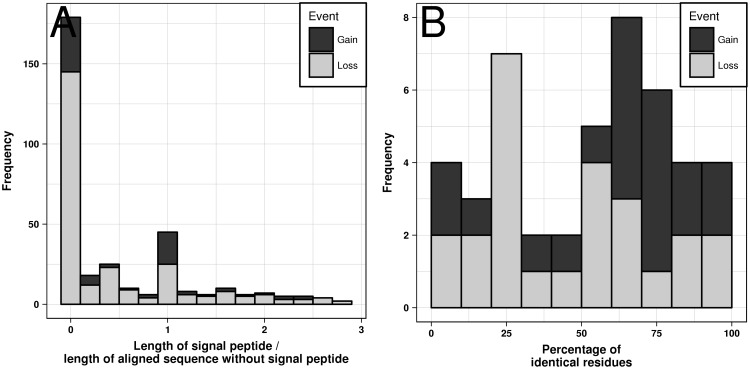
Comparison of signal peptide sequences and the aligned N-terminal sequences without a signal peptide. (*A*) Sequence length ratio. (*B*) Percentage of identical residues for those cases where the length ratio is between 0.9 and 1.1, that is, where both sequences have a comparable length.

How are signal peptides gained and lost, at the molecular level? To answer this question, we analyzed the alignments of extant proteins that descended from their last common ancestor before the gain or loss event, such that some of them contain signal peptides while others do not. Note that only the latest events in the evolutionary sense were taken into account, for example, if a gain event was later on reversed by a loss event, only the loss event was considered. For each alignment associated with a gain or loss event, we calculated the length ratio *lr* between signal peptides and the N-termini devoid of signal peptides, as shown in figure 5*A*. The distribution of *lr* values (fig. 6*A*) points to the existence of two categories of events. The first category, covering 145 loss and 34 gain events, is characterized by *lr* values close to zero, reflecting a full deletion or insertion of an entire signal peptide. An example of such a loss event can be found in the “Pectinesterase” OMA-group 189,619. Pectin methylesterases, found in plant pathogens, play a major role in the first step of soft rot infections. They help to degrade pectin in the plant cell wall, destabilizing it and leading to cell necrosis and tissue maceration. Different plant pathogens have a different inventory of these secreted proteins (Abbott and Boraston 2008). Figure 5*B* shows the alignment of the signal peptide-containing pectin methylesterases (*pemB*) from two Dickeya (former Erwinia) species and four *pemB* orthologs from other Pectobacteria, which lack a signal peptide. Beyond the N-terminal part of the alignment the proteins are highly similar. It should be noted that *pemA*, another pectin methylesterase, does contain a signal peptide in all of these six organisms. The observation that pemB is not exported in all pectin degrading bacteria is in line with an earlier experimental study, which showed that *pemB* is exported in some but not all Dickeya strains (Shevchik et al. 1996). We therefore speculate that, although *pemB* is encoded in all of the Dickeya genomes, its activity may vary dependent on whether or not a signal peptide is present.

We tested the hypothesis that complete deletions and insertions could be caused by transposable elements, but no such elements in proximity to the N-termini of the proteins in the mixed clusters were found by ISEScan (Xie and Tang 2017).

In the second category, covering 25 loss and 20 gain events, proteins with and without signal peptides possess N-terminal amino acid sequences of comparable length. The events are therefore caused by amino acid substitutions, with *lr* values close to one. In most of the cases the N-terminal regions maintain an even higher sequence identity than the average of 52.7% (fig. 6*B*). For example, the gain event alignment of the “putative Invasin” (OMA-group: 83,250) (fig. 5*C*) contains three similar N-terminal sequences, but only one of them possesses a signal peptide. From the six mutations contributing to the difference between the N-termini with and without signal peptides, four mutations strengthen the tripartite structure of a common signal peptide: 1) replacement of threonine by lysine at position four introduces an additional positively charged amino acid, 2) replacement of glycine by alanine at position 22 extends the hydrophobic stretch, and 3) two further mutations affect the cleavage site by changing its sequence from TLA to AMA and thus make it more similar to the canonical AxA motif.

Although the conducted analysis of the mechanism included only the latest events, we were also able to identify 11 mixed clusters where preceding events were reversed. In seven cases, earlier loss events were inverted by a later gain event (“putative lipoprotein,” “hemolysin activator protein,” “RND efflux system outer membrane lipoprotein NodT,” “Fimbrial biogenesis outer membrane usher protein,” “Biofilm PGA synthesis protein pgaA,” and two “Putative uncharacterized proteins”), whereas in two groups a reversal in the opposite direction occurred (“acetyl-coA acetyltransferase” and “secretion monitor”). In the remaining two COGs, the signal peptide was lost, regained, and lost again (“cytochrome b562” and “Soluble lytic murein transglycosylase and related regulatory proteins some contain LysM/invasin domain”).

Our findings indicate that loss events are due to insertions/deletions almost seven times more often than due to mutations. For gain events, this ratio is only 1.5-fold. Indeed, a shift of the gene start will likely delete a signal peptide, whereas a functional signal peptide is not likely to be gained by randomly prepending amino acids to the protein N-terminus. Intuitively, the deletion or mutation of the cleavage site would be the most economical way to disable a signal peptide, but our data do not support this assumption. We calculated sequence identities between the cleavage sites and the remaining N-terminal sequences for protein pairs with and without signal peptide having *lr* values close to one. The Spearman’s rank correlation coefficient between these two sequence identity values is 0.39 for gain and loss events together (*P* = 0.008), 0.49 for loss events (*P* = 0.013), but only 0.22 for gain events (*P* = 0.346), which indicates that the mutation rate in the cleavage sites does not differ from other positions within the signal peptide sequence (see also supplementary fig. S3, Supplementary Material online).

### Functional Classification

We investigated the functional distribution and the localization of the positive/negative and mixed groups based on Gene Ontology annotations (GO-terms) (Ashburner et al. 2000; Gene Ontology Consortium 2015) from three domains: biological process (BP), molecular function (MF), and cellular component (CC). In general, the distribution of GO terms in the mixed clusters is clearly more similar to the one of the positive than in the negative clusters (supplementary fig. S4, Supplementary Material online). COG functions tend to reflect their signal peptide content, with positive and mixed clusters containing significantly more GO terms associated with exported proteins, whereas the negative clusters are mostly associated with intracellular processes, functions, and components. For example, processes involving DNA or RNA, which are localized within the cell, such as “nucleobase-containing compound metabolic process” (GO: 0006139) in the BP category and “nucleotide binding” in the MF category, are prevalent in the negative group. On the other hand, “Cell adhesion” (GO: 0007155), a process which occurs outside of the cell, is almost exclusively found in the positive and mixed groups. The CC categories “outer membrane” (GO: 0019867) and “pilus” (GO: 0009289) are overrepresented in the positive and mixed groups, whereas “intracellular” (GO: 0005622) and “cytoplasm” (GO: 0006737) are more often found in the negative groups. Although the terms in the mixed groups are often similar to those in the positive groups, there are some exceptions, for example, the “aminoglycan metabolic process” (GO: 0006022) from the BP category is prevalent in the mixed groups (in ∼7.6% of its proteins), whereas almost absent in the other two groups (0.8% of the proteins in the negative groups, and 2.1% of the proteins in the positive groups).

### Taxonomy Distribution of Events

For each event, we identified the minimal common taxonomic rank of the descendants of the node where it happened. Gain events preferentially occurred at the order level (32.5%), and somewhat less frequently at the family (28.9%), genus (22.9), and order level (15.7%), whereas loss events occurred mostly at the species level (33.7%) (supplementary table S1, Supplementary Material online). The number of uncertain events increases with the level of the taxonomic rank, from 10.4% at the species level to 60.1% at the order level, mainly because the assignment of a definite signal peptide state gets more difficult toward the root of the tree.

### Symbiotic Relationships and the Loss of Signal Peptides

We investigated the interrelationships between signal peptides, genome sizes, and bacterial lifestyle at two levels: the fraction of signal peptide containing proteins as a function of genome size (fig. 3), and the correlation of signal peptide gain/loss events with the transition from a free-living organism to a commensal organism or an endosymbiont and *vice versa*. It should be noted that these analyses were conducted on our final data set, that is, only with proteins which could be mapped to a COG and have a reliably assigned signal peptide status after the gene start correction, which led to a slightly reduced number of proteins per genome.

In our data set, the 120 free-living bacteria contain on an average 3,596 proteins, compared with 3,730 proteins in the 12 commensals and 1,066 proteins in the 21 endosymbionts. For reference, the average numbers of proteins in the complete genomes of free-living bacteria, commensals, and endosymbionts were 4,511, 4,481, and 1,500, respectively. The Kolmogorov–Smirnov test shows that the protein size distributions between free-living bacteria and commensals are similar (*P* = 0.12), whereas both of them differ significantly from the endosymbiont distribution (*P* = 1.3e^−10^ and *P* = 1.2e^−5^). The same is true for the percentage of proteins containing signal peptides, with the average numbers being 9.5% for the free-living bacteria, 10.0% for the commensals, and 2.8% for the endosymbionts. Again, the distributions are significantly different when comparing free-living bacteria or commensals against endosymbionts (*P* = 5.8e^−11^ and *P* = 3.8e^−6^), whereas being similar between the latter two (*P* = 0.18). The same holds true according to the two sample Cramér–von Mises test calculated for the multivariate distributions of protein sizes and fractions of signal peptides between the three classes (*P* values close to zero between free-living/commensal and endosymbionts; *P* = 0.26 for free-living and commensals).

Symbionts tend to have reduced genomes as a consequence of losing genes whose functions are delegated to the host organism. As a result of genomic shrinkage, a larger proportion of the remaining genes is involved the basic cellular functions, such as replication, transcription, and translation, whereas many less essential functions, including those associated with amino acid synthesis or other metabolic processes, which can be provided by the partner or host may be lost (Andersson and Kurland 1998). We calculated a discrimination score *d(a, b, g)* for each COG *g* (see Materials and Methods) to evaluate whether or not the possession of a signal peptide is a sufficiently discriminative characteristic for telling apart endosymbionts (endo), commensals (com), and free-living bacteria (fl). Out of the 440 mixed groups, 182 contained at least one free-living bacterium and at least one endosymbiont, 104 at least one commensal and at least one endosymbiont, and 221 contained at least one free-living bacterium and at least one commensal. According to the two-tailed Fisher’s exact test discrimination between endosymbionts and free-living bacterial was significant in seven groups, of which the following six yielded *d(fl, endo, g)* scores >0 (supplementary fig. S5, Supplementary Material online), indicating an association of the signal peptide-less proteins with endosymbionts: “flagellar biosynthetic protein flip,” “endonuclease I,” “mechanosensitive ion channel,” “d-alanyl-d-alanine carboxypeptidase,” “ErfK/YbiS/YcfS/YnhG family protein,” and “N-acetylmuramoyl-L-alanine amidase.” We found only one COG (“Spore coat U domain protein”) with a significant discrimination and a *d(fl, endo, g)* <0, indicating that signal peptides preferentially occur in the proteins from symbiotic bacteria rather than in free-living organisms. In three out of the 104 COGs containing both endosymbionts and commensals the signal peptide state was significantly associated with the lifestyle. We found two groups with *d(com, endo, g)* >0 (“putative transferase” and “mechanosensitive ion channel”), as well as one <0 (“tonB-system energizer ExbB”). Comparing the groups containing free-living and commensals, there were also three significant groups, two with a *d(fl, com, g)* >0 (“Putative uncharacterized protein,” “peptidase M15D vanX D-ala-D-ala dipeptidase”) and one <0 (“putative transferase”). The Spearman’s rank correlation coefficient of 0.74 between all *d(fl, endo, g)* and *d(com, endo, g)* scores is highly significant (*P* = 2.2e^−16^), reflecting resemblance in genome size and signal peptide content of free-living bacteria and commensals. The overall distribution of significant *d(a, b, g)* scores (supplementary fig. S5, Supplementary Material online) indicated that signal peptides are a discriminating feature between endosymbionts and free-living bacteria or commensals.

We analyzed the GO annotations of the individual proteins with or without signal peptides in the mixed clusters (supplementary fig. S6, Supplementary Material online). With regard to cellular component (CC) nonsecreted proteins are preferably tagged as “cytoplasm” (GO: 0005737), whereas the secreted ones are annotated with “membrane” (GO: 0016020) which includes “outer membrane” (GO: 0019867), “periplasmic space” (GO: 0042597) and similar terms. In the MF and BP categories proteins containing a signal peptide are involved in “channel activity” (GO: 0015267) and “transport” (GO: 0006810), whereas those without a signal peptide take part in “nucleotide binding” (GO: 0000166) and “carboxylic acid biosynthetic process” (GO: 0046394).

Although the previous analysis was conducted for all bacteria in our data set, we additionally compared GO-term annotations of proteins with and without a signal peptide for each lifestyle separately and found that functional assignments generally do not correlate with the lifestyle, with few exceptions. Some GO-terms are more (MF: “nucleotide binding”) or less (CC: “membrane”) frequently associated with endosymbionts compared with free-living bacteria and commensals (supplementary fig. S6, Supplementary Material online).

Assuming that some species may have changed their lifestyle in the course of evolution, we conducted an additional parsimony analysis using the endosymbiont/commensal/free-living annotations together with the signal peptide events (table 2). The proportions of gain/loss events are similar for all transitions to any lifestyles, for example, 1.1% of the transitions to endosymbionts are accompanied by a loss event but only 0.4% by gain events. However, dependent on the nature of a transition there is a noticeable difference in the number cases where signal peptide assignments remain negative: this applies to 28.7% of the transitions to endosymbionts, but only to 19.8% and 15.6% of the transitions to free-living bacteria and commensals, respectively. We speculate that in many such cases the loss of the signal peptide might not have happened simultaneously with the transition to a specific lifestyle, but rather before or after it. Qualitatively, this apparent difference seems to strengthen our conjecture, but it fails to reach statistical significance as the number of such events is quite low compared with the total number of events in our analysis.

**Table 2:** Contingency Table of Signal Peptide Gain and Loss Events and Their Correlation with Changes of Bacterial Lifestyles

Event	Transition to Free-Living Bacterium	Transition to Endosymbiont	Transition to Commensal	Uncertain Transition	Total Number of Signal Peptide Events
**Gain**	76 (0.4%)	2 (0.4%)	2 (0.1%)	3 (0.9%)	83
**Loss**	263 (1.3%)	5 (1.1%)	14 (0.9%)	6 (1.7%)	288
**Uncertain**	268 (1.3%)	15 (3.3%)	5 (0.3%)	58 (16.9%)	346
**Keep signal peptide**	15,581 (77.2%)	298 (66.4%)	1,312 (83.0%)	227 (66.2%)	17,418
**Stay without signal peptide**	4,006 (19.8%)	129 (28.7%)	247 (15.6%)	49 (14.3%)	4,431
**Total number of transition events**	20,194 (100%)	449 (100%)	1,580 (100%)	343 (100%)	

## Conclusions

Computational prediction of signal peptides is an indispensable step in bacterial genome annotation, but their evolutionary dynamics has not been comprehensively studied. We investigated the gain and loss patterns of signal peptides between orthologous proteins from *Enterobacterales* and found that 1.9% of COGs contain proteins both with and without signal peptides. Reconstruction of ancestral signal peptide states by parsimony analysis in such mixed groups clearly indicates that signal peptides get lost more often in the course of evolution than they are gained. We also show that signal peptide gains tend to be more ancient events, predominantly occurring at the family and probably at the order level, although a high number of uncertain events at this latter level makes it impossible to draw definitive conclusions. At the same time, signal peptide losses might be more recent events as we found most of them at the species level. Gain and loss events occur by either a complete insertion or deletion of the entire signal peptide sequence or by retaining the N-terminal sequence and mutating residues to enable or disable the signal peptides functionality. The prevalent loss of signal peptides is accompanied by genome reduction, with smaller genomes of endosymbiotic bacteria containing a lower percentage of signal peptides than free-living and commensal bacteria. In some enterobacterial COGs the presence or absence of a signal peptide alone is sufficient to discriminate between endosymbionts, on the one hand, and free-living bacteria or commensals, on the other hand. Finally, we demonstrate that signal peptide loss events preferentially occur in the course of transition from free-living bacteria/commensals to endosymbionts.

## Supplementary Material

Supplementary data are available at *Genome Biology and Evolution* online.

## Supplementary Material

Supplementary Figures and Tables
