# Supplementary material for: Evolutionary Interplay between Symbiotic Relationships and Patterns of Signal Peptide Gain and Loss
Source: Genome Biol Evol. 2018 Mar 19;10(3):928–38. doi: 10.1093/gbe/evy049 (PMC5952966; doi:10.1093/gbe/evy049)
Supplement: Supplementary Figures and Tables [file evy049_supp.pdf]

1 SUPPLEMENTARY TABLES

Table S1: Taxonomic rank of signal peptide gain, loss and uncertain events.

Event	Species	Genus	Family	Order	Total
**Gain**	13 (15.7%)	19 (22.9%)	24 (28.9%)	27 (32.5%)	83
**Loss**	97 (33.7%)	45 (15.6%)	93 (32.3%)	53 (18.4%)	288
**Uncertain**	36 (10.4%)	18 (5.2%)	84 (24.3%)	208 (60.1%)	346

2 SUPPLEMENTARY FIGURES

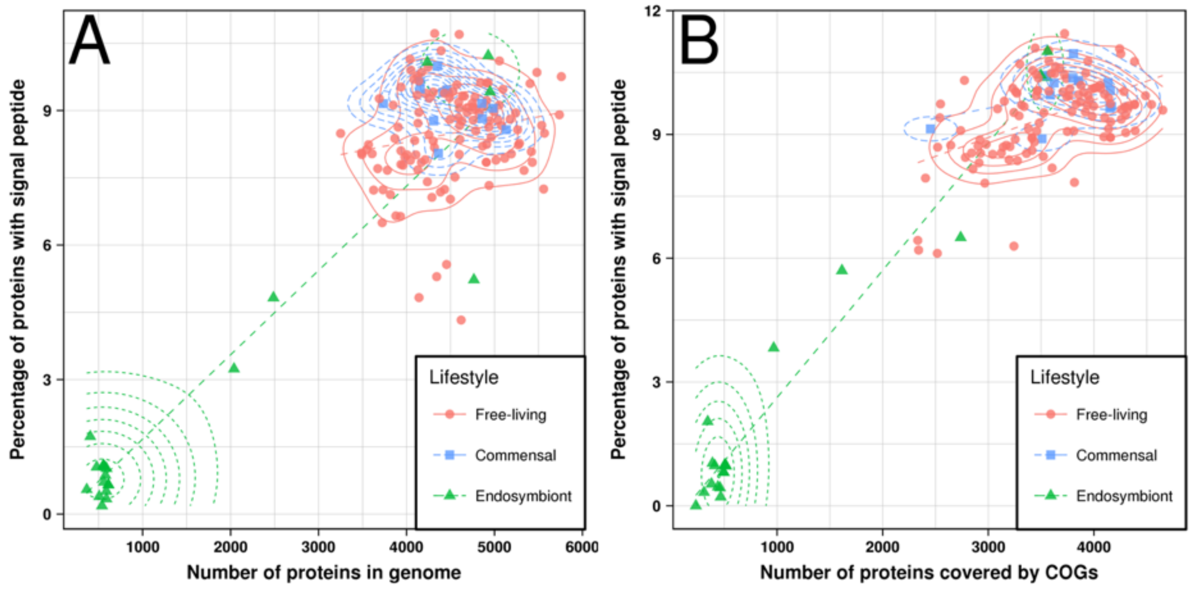

Figure S1: Number of proteins in a genome vs. the percentage of proteins that possess a signal peptide (A) using the full dataset, and (B) after mapping of the proteins to COGs. In addition to the raw values, the two-dimensional density and a linear fit (dashed lines) for each lifestyle is shown.

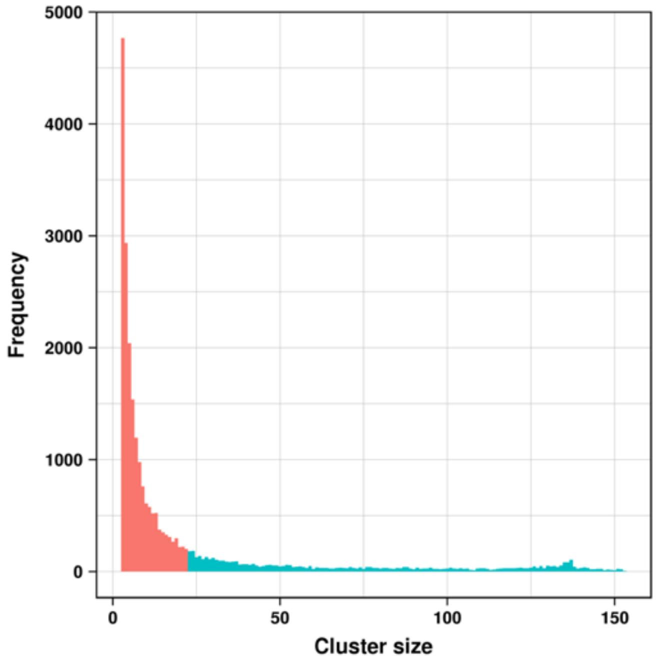

Figure S2: Distribution of cluster sizes. Histogram bins containing clusters that are smaller and larger than the average cluster size are colored red and green, respectively.

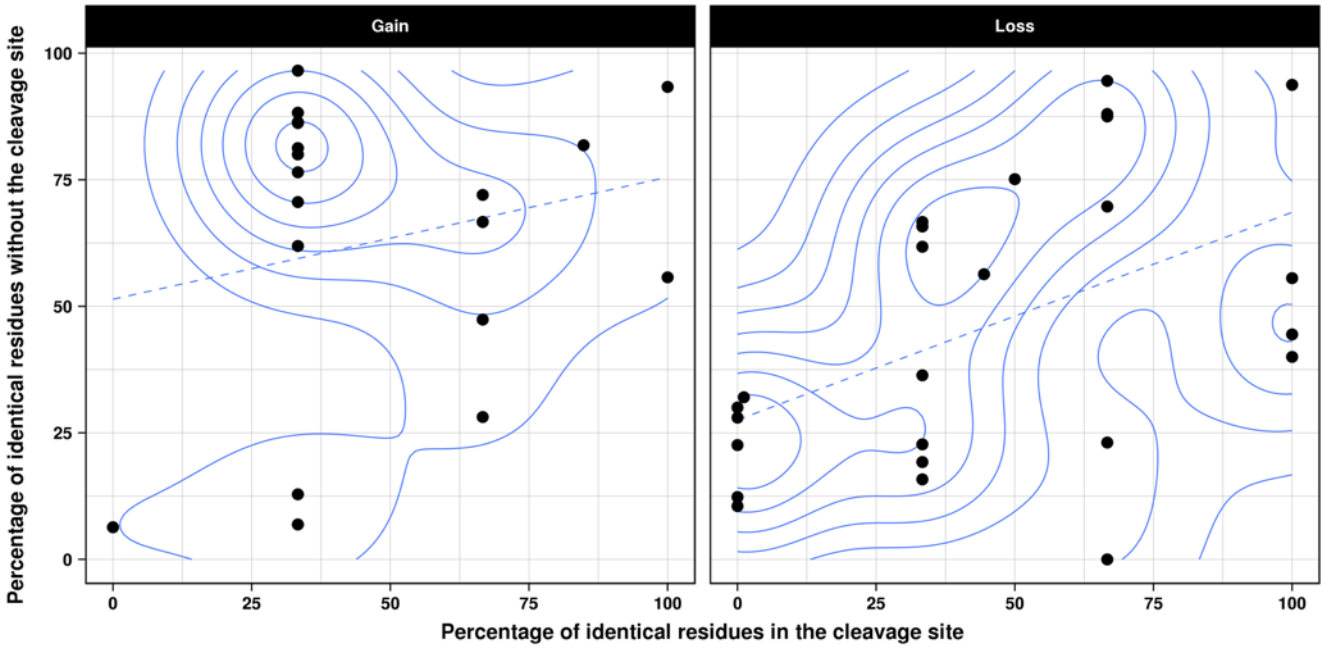

Figure S3: Identity between signal peptide sequences and the aligned N-terminal sequences without a signal peptide according to the sequence identity at the cleavage site and the remaining positions separated by gain and loss eve

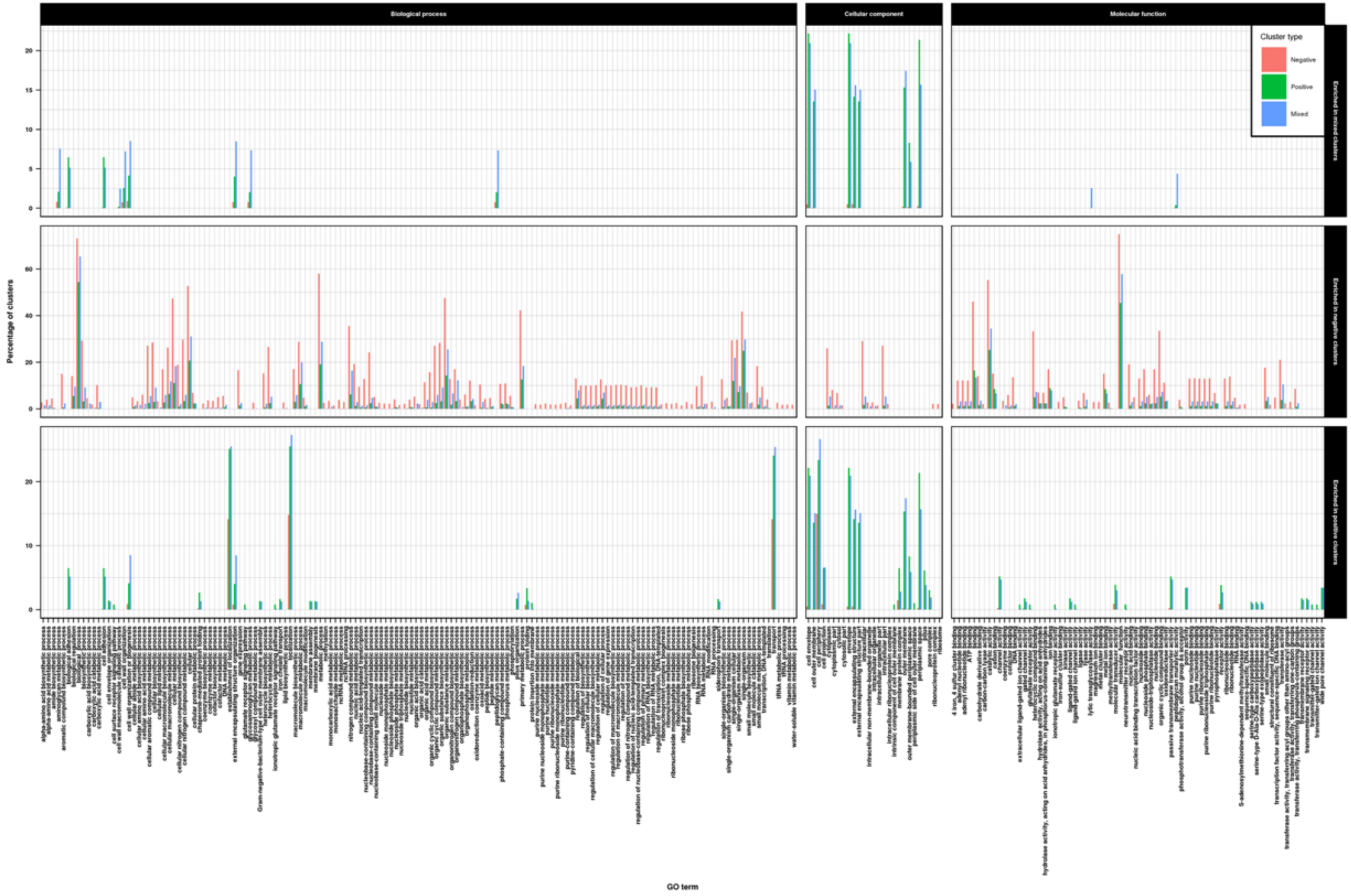

Figure S4: Percentage of proteins having a specific enriched GO-term for the mixed (first row), negative (second row) and positive (third row) groups. The columns match the three ontologies, biological process, cellular component and molecular function.

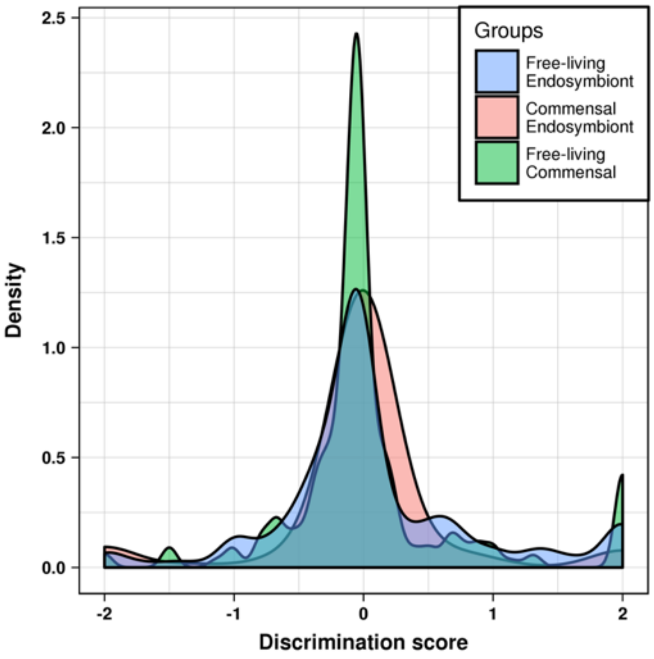

Figure S5: Density plot of discrimination scores between different lifestyles of bacteria.

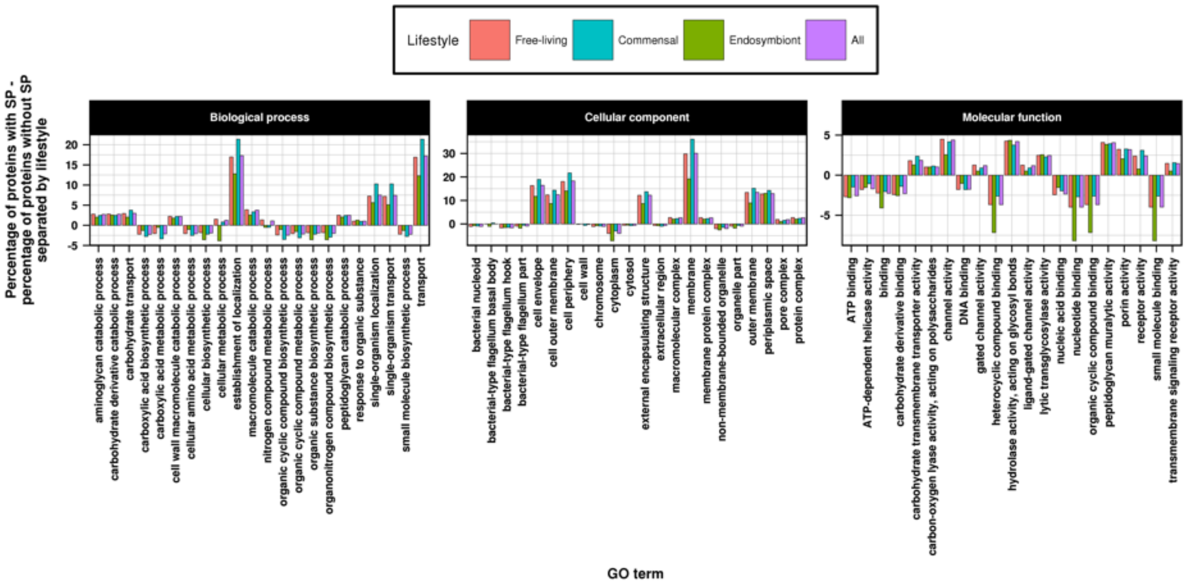

Figure S6: Percentage of proteins without signal peptide subtracted from the percentage of proteins with signal peptide in the mixed clusters having a specific enriched GO-term and belonging to organisms with a certain lifestyle.
